# Dietary fats modify vascular fat composition, eNOS localization within lipid rafts and vascular function in obesity

**DOI:** 10.14814/phy2.13820

**Published:** 2018-08-14

**Authors:** Daniel W. Nuno, Lawrence J. Coppey, Mark A. Yorek, Kathryn G. Lamping

**Affiliations:** ^1^ Department of Internal Medicine Roy J. and Lucille A. Carver College of Medicine University of Iowa Iowa City Iowa; ^2^ Iowa City Veterans Affairs Healthcare System Iowa City Iowa; ^3^ Department of Pharmacology Roy J. and Lucille A. Carver College of Medicine University of Iowa Iowa City Iowa

**Keywords:** Caveolae, caveolin‐1, cyclooxygenase, lipid raft, nitric oxide synthase

## Abstract

We tested whether dietary fatty acids alter membrane composition shifting localization of signaling pathways within caveolae to determine their role in vascular function. Wild type (WT) and caveolin‐1‐deficient mice (cav‐1 KO), required for vascular caveolae formation, were fed low fat (LF), high saturated fat (HF, 60% kcal from lard), or high‐fat diet with 50:50 lard and n‐3 polyunsaturated fatty acid‐enriched menhaden oil (MO). HF and MO increased body weight and fat in WT but had less effect in cav‐1 KO. MO increased unsaturated fatty acids and the unsaturation index of aorta from WT and cav‐1 KO. In LF WT aorta, endothelial nitric oxide synthase (eNOS) was localized to cav‐1‐enriched low‐density fractions which shifted to actin‐enriched high‐density fractions with acetylcholine (ACh). HF and MO shifted eNOS to high‐density fractions in WT aorta which was not affected by ACh. In cav‐1 KO aorta, eNOS was localized in low‐density non‐caveolar fractions but not shifted by ACh or diet. Inducible NOS and cyclooxygenase 1/2 were not localized in low‐density fractions or affected by diet, ACh or genotype. ACh‐induced dilation of gracilis arteries from HF WT was similar to dilation in LF but the NOS component was reduced. In WT and cav‐1 KO, dilation to ACh was enhanced by MO through increased role for NOS and cyclooxygenase. We conclude that dietary fats affect vascular fatty acid composition and membrane localization of eNOS but the contribution of eNOS and cyclooxygenase in ACh‐mediated vascular responses is independent of lipid rafts.

## Introduction

The health implications of excess weight and obesity which continue to increase world‐wide include development of Type 2 diabetes and cardiovascular disease (Collaborators et al. 2017). Obesity is primarily a consequence of increased fat mass following excess nutrient consumption often from a diet high in saturated fats. Optimizing dietary fat composition is a target for reducing obesity and its related disease burden, but the appropriate substitute for saturated fats in the diet is controversial. Clinical trials controlling fat consumption have not demonstrated clear benefits of monounsaturated (MUFAs) versus either n‐3 or n‐6 polyunsaturated fatty acids (PUFAs) in reversing cardiovascular disease (Degirolamo and Rudel 2010; Gillingham et al. 2011). We previously examined effects of short‐term substitution of a portion of the dietary saturated fatty acids with n‐3 PUFAs as a model of a recommended change in dietary fats in established obesity (Lamping et al. 2013). High‐fat diet increased circulating levels of glucose, insulin, cholesterol, and leptin. Substituting half of the saturated fats with n‐3 PUFA enriched menhaden oil (MO) reduced circulating cholesterol, insulin and leptin levels along with improved vascular function in mice better than diets enriched in n‐6 PUFAs or MUFAs (Lamping et al. 2013). Levels of circulating triglycerides and total fatty acids did not change. While vascular function was improved with substitution of dietary saturated fats with n‐3 PUFAs, the mechanisms underlying the benefits were not clear.

Mediators of acetylcholine (ACh)‐mediated vasodilation depend on animal species, tissue, sex, and vessel size with varying contributions from nitric oxide (NO) from nitric oxide synthase (NOS), prostanoids from cyclooxygenase (Cox) and endothelial‐dependent hyperpolarizing factor. The balance of these vasodilating mediators can shift with disease and is modulated in dietary‐ and genetically induced obesity (Guo et al. 2005; Bagi 2009; Salvemini et al. 2013). Dietary fats may shift the contribution from constitutive endothelial NOS (eNOS) and Cox‐1 to inducible forms of NOS (iNOS) and Cox‐2. Both eNOS and Cox‐1 are involved in the regulation of physiological responses whereas iNOS and Cox‐2 generally play a greater role in inflammatory and pathophysiological conditions.

We previously examined effects of dietary fatty acids on responses to ACh. Dilation in response to ACh was modestly depressed in small arteries perfusing the gracilis muscle from mice on high saturated fat (HF) diet compared to arteries from mice on low‐fat (LF) diet (Lamping et al. 2013). Substitution of a portion of the saturated fatty acids with n‐3 PUFA MO improved responses to ACh. Mechanisms involved in the beneficial effect of n‐3 PUFA were not examined. A potential impact of dietary fatty acids on vascular function may involve effects on the plasma membrane. Normally, eNOS localizes to specialized lipid rafts, caveolae, where it binds to caveolin‐1 (cav‐1) to inhibit its activity (Garcia‐Cardena et al. 1996; Liu et al. 1997; Drab et al. 2001; Fulton et al. 2002). In cultured cells, n‐3 PUFAs affects plasma membrane lipid levels and formation of caveolae and non‐caveolar lipid rafts (Ma et al. 2004; Li et al. 2007a, 2007b; Ye et al. 2010). We have previously used mice deficient in cav‐1 to examine the contribution from caveolae to vascular responses to serotonin (Nuno et al. 2012). In the absence of cav‐1, caveolae cannot assemble but not non‐caveloar lipid rafts remain. Comparing effects in the presence and absence of cav‐1 provides one approach for determining the contribution of caveolae versus non‐caveolae lipid rafts in vascular responses. In this study, we focused on whether dietary n‐3 fatty acids affect membrane composition and/or localization of signaling pathways, specifically eNOS and Cox, in vasculature to modify vascular responses. We proposed that dietary fatty acid saturation may differentially impact membrane localization of signaling pathways within caveolar and non‐caveolar lipid rafts affecting their contribution to vascular responses. As the contribution from eNOS declines with high saturated fat diet, the role of other signaling pathways, namely cyclooxygenase, increases (Szerafin et al. 2006; Bagi 2009) which may be related to localization within lipid rafts.

## Methods

### Animal model

The animal protocol was reviewed and approved by the Animal Care and Use Committee of the Iowa City Veterans Affairs Health Care System and complied with the Guiding Principles for Research Involving Animals and Human Beings. Beginning at 9 weeks of age, male C57BL/6J mice (wild type, WT) and mice deficient in caveolin‐1 (B6.Cg‐*Cav1* ^*tm1Mls*^/J, Jackson Laboratories, stock #007083, cav‐1 KO) were randomly fed either normal mouse diet (low fat, LF, 13% kcal fat, 7001; Teklad, Harlan Labs, Madison, WI) or high saturated fat diet (HF, 60% kcal fat from lard, Research Diets, New Brunswick, NJ, D12492) for 12 weeks. To determine if a change in dietary fatty acids could reverse the effects of a high saturated fat diet, at the end of 12 weeks in a random group of mice on high‐fat diet, half of the kcal from lard was replaced with menhaden oil (MO, Research Diets, New Brunswick, NJ, D10122003) and the mice were maintained for an additional 6–10 weeks. Prior to terminal studies, body composition (nuclear magnetic resonance Bruker LF90II) was measured. For terminal study, mice were euthanized with ketamine/xylazine (100/10 mg/kg, ip), weighed, and tissues and serum obtained for measurement of vascular function, protein expression, glucose and lipid analysis.

### Assessment of vascular function

The gracilis muscle was removed and placed in ice cold Krebs buffer (mmol/l: NaCl 118, KCl 4.7, CaCl_2_ 2.5, MgSO_4_ 1.2, KH_2_PO_4_ 1.2, NaHCO_3_ 25, glucose 5). Gracilis arteries (diameter range 72–186 *μ*m) were isolated, cannulated onto glass micropipettes filled with physiological salt solution in an organ chamber and secured with suture before being pressurized to 60 mmHg pressure. Warmed (37°C), oxygenated (20% O_2_, 5% CO_2_, and 75% N_2_) modified Krebs solution was continuously circulated through the chamber as the arteries were equilibrated for 45–60 min prior to study. Vessel lumen diameters were measured with a video microscopy system and an electronic dimension analyzer. Vessel viability was assessed by a minimum of 50% constriction in response to KCl (50 mmol/L). For assessment of vascular relaxation, arteries were contracted with thromboxane mimetic, U46619 (50 to 60% of maximal, 0.05 to 0.1 *μ*g/mL) before concentration response curves to acetylcholine (ACh, 1 nmol/L–10 *μ*mol/L) and nitroprusside (SNP, 1 nmol/L–10 *μ*mol/L) were measured. Responses were measured before and after inhibition of nitric oxide synthase with nitro‐L‐arginine (LNNA, 100 *μ*mol/L) or cyclooxygenase with indomethacin (INDO, 10 *μ*mol/L) for a minimum of 30 min.

### Vascular lipid analysis

Three to four aortae from mice were pooled to analyze fatty acid composition using gas‐liquid chromatography (Yorek et al. 1984a, 1984b; Lamping et al. 2013). Lipids were extracted from aortae with a 2:1 (vol:vol) mix of chloroform and methanol followed by phase separation with a solution of NaCl (154 mmol/L) and HCl (4 mmol/L). The lipid fraction was transesterified in 14% boron trifluoride in methanol and fatty acid methyl esters extracted into heptane before separation by gas‐liquid chromatography (Yorek et al. 1984a, 1984b; Lamping et al. 2013). Individual fatty acid peaks as % of total fatty acids were identified by comparison to known fatty acid standards.

### Isolation of low‐density lipid rafts

Thoracic aortae were isolated, cleaned of adherent fat and mounted on metal rings connected to a force transducer in an organ bath filled with oxygenated warmed Krebs solution. Following equilibration where tension was increased to 0.75 g, vessels were flash frozen under basal conditions or following contraction with U46619 and relaxation with ACh (1 *μ*mol/L). Three to four aortae were pooled for each lipid raft preparation.

Lipid raft fractions were isolated using the detergent‐free sucrose density centrifugation method with sodium carbonate buffer developed by Song et al. (1996). Samples were dounced in liquid nitrogen before addition of NaCO3 (500 mmol/L, pH 11) containing complete protease inhibitor cocktail (Roche) and phosphatase inhibitors (Sigma‐Aldrich). Samples were sonicated (50% power, 10 sec, three times) and centrifuged (7500 × g, 5 min at 4°C). An aliquot of the supernatant was adjusted to 45% sucrose and layered onto a sucrose gradient of 35% and 5% sucrose solutions [0.25 mM NaCO3 in 24 mmol/L 2‐(N‐morpholino)‐ethanesulfonic acid, at pH 6.5 and 150 mmol/L NaCl]. Samples were centrifuged for 20–24 h, at 200,000 × g, 4°C. Equal fractions of the gradient were collected from lightest (fraction 1) to heaviest (fraction 9) for Western immunoblotting.

### Western immunoblotting

Protein expression was measured in whole cell lysates of gracilis arteries as well as lipid raft fractions of aorta using western immunoblotting. For whole cell lysates only, arteries were flash frozen, dounced in liquid nitrogen, a denaturing lysis buffer added (Tris‐HCl 50 mmol/L, EDTA 0.1 mmol/L, EGTA 0.1 mmol/L, SDS 0.1%, NP40 1%, deoxycholic acid 2.4 mmol/L, protease and phosphatase inhibitors) and samples were sonicated three times on ice. Samples were centrifuged and protein concentration determined using the bicinchoninic acid method.

Equal aliquots of each lipid raft fraction or equal amounts of whole cell lysate protein were subjected to SDS PAGE analysis. After blocking, immunoblotting was performed with antibodies to eNOS (1:500, BD Transduction, #160297), p‐eNOS Ser1177 (1:250, Cell Signaling, #9571), iNOS, (1:500, Boster, #PA1330‐1), Caveolin‐1 (1:500, BD Sciences, #610407), cyclooxygenase‐1 (1:500, Boster, #M00811), cyclooxygenase‐2 (1:500, Boster, #M00084), and *β*‐actin (1:600, Sigma‐Aldrich, #A2228) followed by secondary antibodies conjugated to horseradish peroxidase and analyzed using Image J (NIH). Protein expression in whole cell lysate was normalized to actin. Protein expression in lipid raft fractions was expressed as a percentage of the total in all fractions from low‐density fractions to heavy density fractions.

### Statistical analysis

Data were analyzed using PRISM and are presented as mean ± SE. Comparisons of body weight and composition and aortic fatty acid concentration were compared using one way ANOVA followed by Tukey's for multiple comparisons. All n's for lipid rafts and Western immunoblots represent the number of pooled samples. Western immunoblots of whole cell lysate were compared using two‐tailed unpaired student's *t* test or ANOVA for comparison of multiple groups. Protein expression in lipid rafts was compared using two‐tailed unpaired t tests. Concentration response curves for vascular responses were compared using repeated measures two‐way ANOVA followed by Sidak's or Tukey's for multiple comparisons. Significance was defined as *P* ≤ 0.05.

## Results

### Effect of diet on body weight, body composition, and glucose level

**Figure 1:**
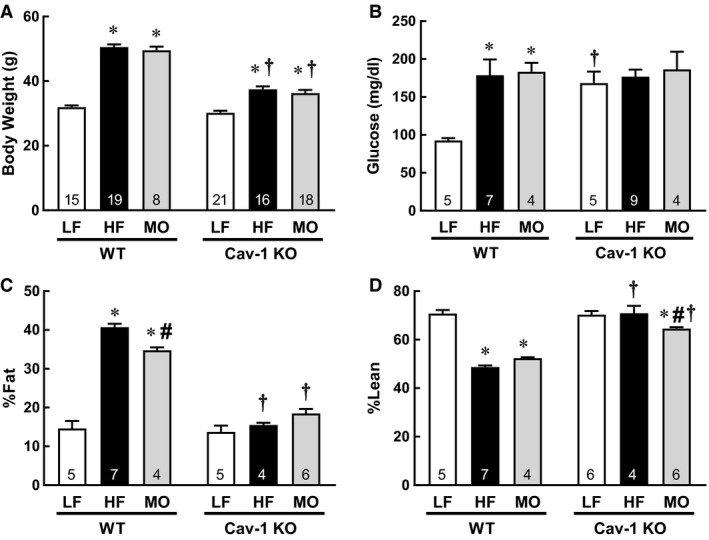
(A) Body weight (g), (B) fasting glucose level (mg/dl), (C) % Fat, and (D) % Lean in WT and Cav‐1 KO mice on LF, HF, and MO diets. Mean ± SEM, n per group in bar, *P < 0.05 versus LF within same genotype, #*P* < 0.05 versus HF within same genotype, t *P* < 0.05 versus WT on same diet. ANOVA with Tukey's for multiple comparisons.

Body weight was increased similarly in WT mice on HF and MO diets compared to normal LF (Fig. 1A). Body fat mass and %fat were less in MO‐fed mice compared with HF but both were greater that LF mice (WT 4.6 ± 0.7 g, HF 20.0 ± 0.5 g*, MO 16.4 ± 0.3 g*#, *P < 0.05 vs. LF, #*P* < 0.05 vs. HF; %fat Fig. 1C, *n* = 4–7). Lean body mass did not differ in WT mice on either high‐fat diet which resulted in a reduction in the %lean in both groups compared to LF (LF 21.9 ± 0.4 g, HF 24.0 ± 0.6 g, MO 24.7 ± 0.8 g, % lean Fig. 1D). Although cav‐1 KO mice on HF and MO gained similar amounts of weight, the weight gain in both groups was less than in WT on the same diet (Fig. 1A). Only MO diet significantly increased the amount of fat in cav‐1 KO mice but the increase was modest compared to WT mice (LF4.1 ± 0.7 g, HF 6.0 ± 0.2 g, MO 7.4 ± 0.3 g*, *P < 0.05 vs. LF; %fat Fig. 1C, *n* = 4–6). Also in contrast to WT mice, the amount of lean mass significantly increased in cav‐1 KO mice on both HF and MO diets (LF 21.8 ± 0.4 g, HF 27.5 ± 0.5 g*, MO 26.0 ± 0.9 g*, *P < 0.05 vs. LF). As %of body weight, there was a slight reduction in %lean in cav‐1 KO on MO (Fig. 1D). Fasting glucose levels were increased similarly by HF and MO compared with LF WT mice (Fig. 1B). Fasting glucose levels were increased in all cav‐1 KO mice compared with WT irrespective of diet (Fig. 1B). Thus, high‐fat diets containing all saturated fats or an equivalent combination of saturated and n‐3 PUFA contributed to obesity and elevated fasting glucose levels. Cav‐1 deficiency diminished effects of high‐dietary fats on body weight primarily by preventing an increase in body fat despite no improvement in fasting glucose.

### Effect of diet on vascular fatty acid composition

**Figure 2:**
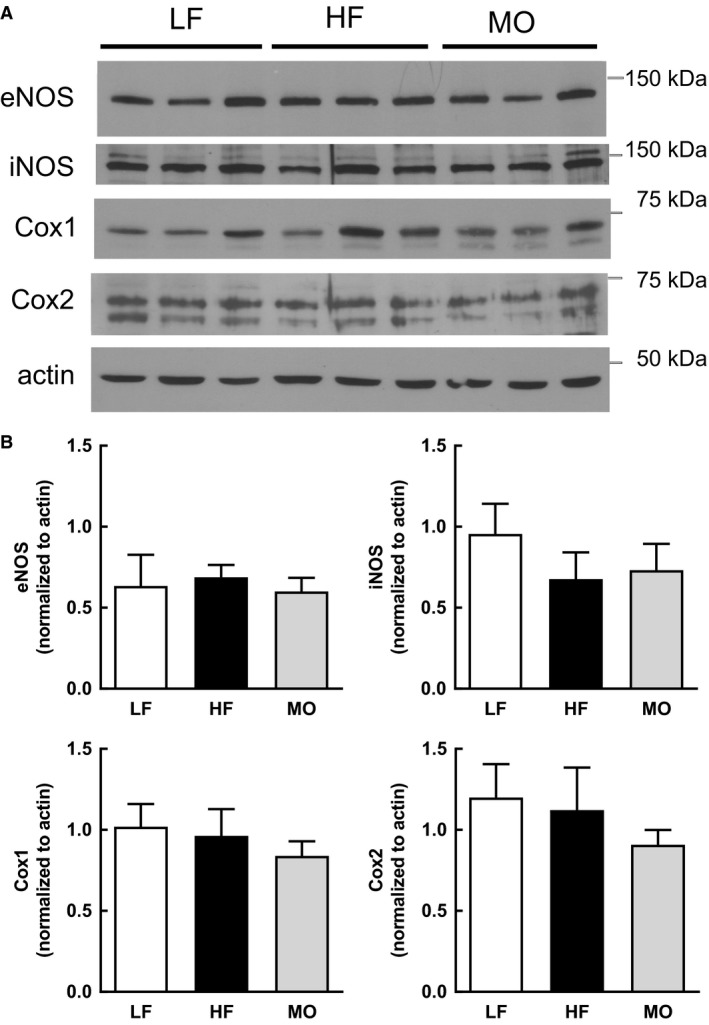
Fatty acid composition (% of Total) and unsaturation index of aorta from WT and cav‐1 KO mice on LF (WT *n* = 6, cav‐1 KO *n* = 4), HF (WT *n* = 5, cav‐1 KO *n* = 3) and MO diets (WT *n* = 6, cav‐1 KO *n* = 4). Mean ± SEM, *P < 0.05 versus LF and #*P* < 0.05 versus HF within genotype. ANOVA with Tukey's for multiple comparisons.

We have previously reported that both liver and muscle fatty acid composition are modified by dietary fats (Lamping et al. 2013). To determine whether diet also affects vasculature, we compared fatty acid composition of aorta from WT and cav‐1 KO mice. HF diet had minimal effects on fatty acid composition of aorta from WT mice with only a reduction in %16:1 (Fig. 2C). In aorta of WT mice, MO increased levels of 20:5 eicosapentanoic acid (EPA, Fig. 2F) and 22:6 docosahexaenoic acid (DHA, Fig. 2H) and reduced percentage of 18:1 and 18:2 (Fig. 2D and E) which increased the unsaturation index (Fig. 2I). Similar to WT mice, HF diet had minimal effects on fatty acid composition of aorta from cav‐1 KO mice. In cav‐1 KO mice, HF significantly increased %18:2 (Fig. 2E). MO shifted the fatty acid distribution in cav‐1 KO aorta similar to WT: levels of 18:3, 22:4, and 22:6 increased and 18:1 decreased which increased the unsaturation index (Fig. 2). Thus, dietary fatty acids affect vascular fatty acid composition. Surprisingly, although HF and MO diets had modest effects on body weight and percent body fat in cav‐1 KO mice, these diets shifted the fatty acid composition of aorta similar to effects in WT mice. These changes in vascular fatty acid composition could impact lipid raft and/or caveolae composition to modify localization of signaling pathways involved in the regulation of vascular reactivity.

### Effect of dietary fatty acids on NOS and Cox expression and localization

**Figure 3:**
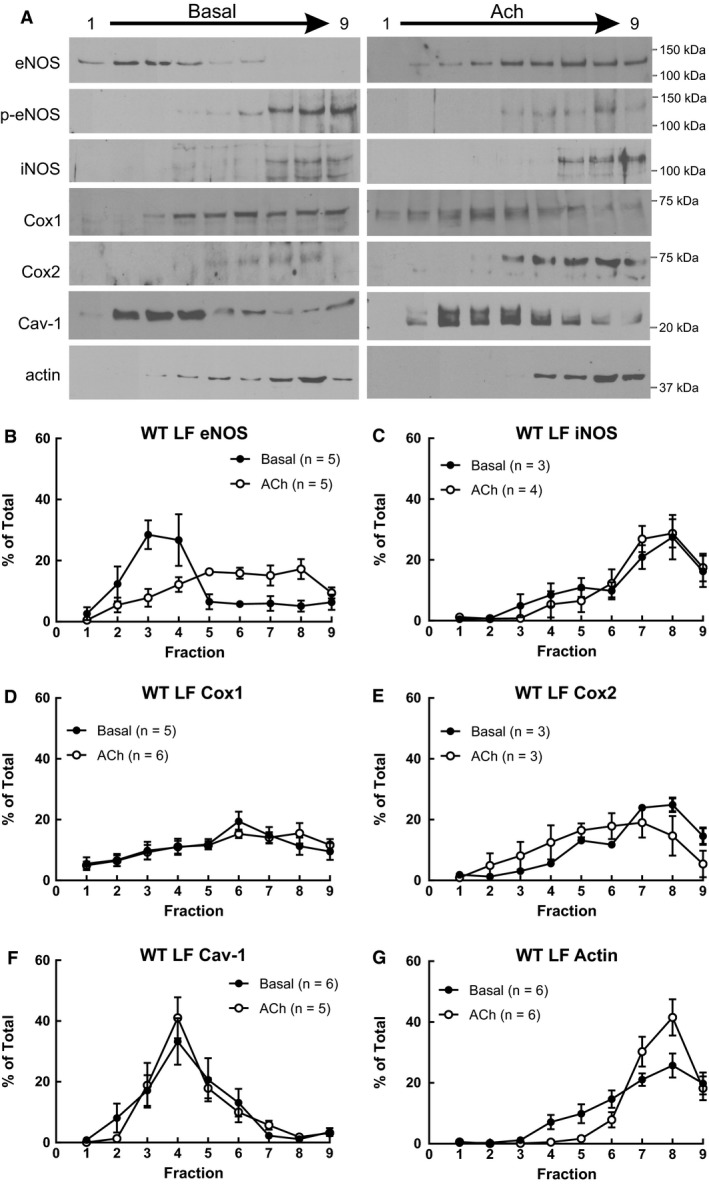
(A) Representative western immunoblots of eNOS, iNOS, Cox1, Cox2, and actin in whole cell lysate from gracilis arteries from WT mice on LF, HF, and MO (*n* = 3 each). (B) Expression of eNOS, iNOS, Cox1, and Cox2 normalized to actin (Mean ± SEM,* n* = 6 each). There were no significant differences in expression of any protein with diet. One‐way ANOVA.

**Figure 4:**
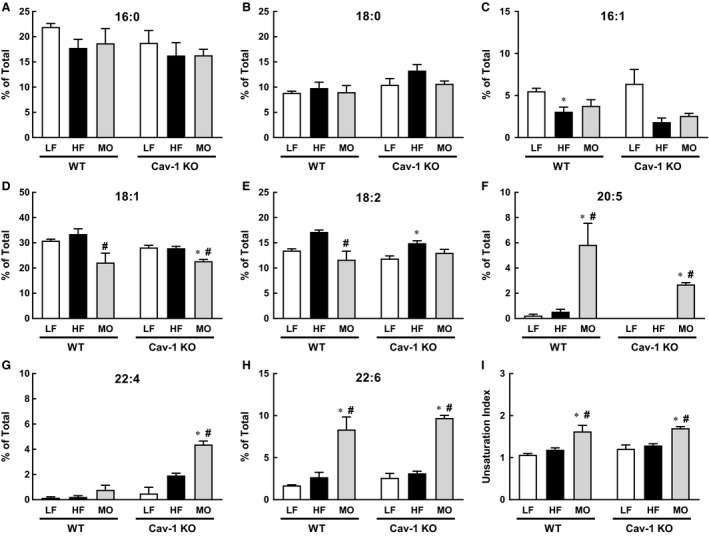
(A) Representative western immunoblots of eNOS, Ser1177 p‐eNOS, iNOS, Cox1, Cox2, Cav‐1, and actin in lipid raft fractions from WT LF aorta under basal conditions and following relaxation to ACh (1 *μ*mol/L). Only eNOS was localized to low‐density cav‐1 containing fractions under basal conditions which shifted to high‐density fractions in ACh‐stimulated aorta. Expression of eNOS (B), iNOS (C), Cox1 (D), Cox 2 (E), Cav‐1 (F), and actin (G) as a percent of total in low‐density fractions (1) to heavy density fractions (9) of aorta from WT mice on LF diet under basal conditions or following relaxation to acetylcholine (ACh, 1 *μ*mol/L). eNOS was primarily localized to cav‐1 containing low‐density fractions under basal conditions which shifted to heavy fractions with ACh. iNOS, Cox1, and Cox2 were not localized to low‐density cav‐1 containing fractions or affected by ACh. The percent of actin in heavy density fractions increased with ACh (G). Mean ± SEM. Two‐tailed unpaired *t* test.

We determined the impact of diet on expression of eNOS, iNOS, Cox1, and Cox2 in whole cell lysate of gracilis arteries from WT mice. Neither high saturated fat nor MO‐enriched diet affected expression of eNOS, iNOS, Cox1 or Cox2 in gracilis arteries (Fig. 3). As dietary fatty acids altered vascular fatty acid composition which could impact membrane lipids, we determined whether diet modified localization of NOS or Cox within lipid rafts of aorta and whether localization was shifted with ACh (1 *μ*mol/L). Under basal conditions in aorta from WT mice on LF, only eNOS was localized in low‐density fractions where cav‐1 was also found (Fig. 4). iNOS was localized to high‐density fractions where actin was localized (Fig. 4). Cox1 and Cox2 were uniformly distributed throughout the gradient (Fig. 4). With ACh stimulation, the most prominent change was the distribution of eNOS. In contrast to its localization in low‐density fractions under basal conditions, eNOS was shifted to high‐density fractions with ACh (Fig. 4A and B, % of total in fractions 2–5: Basal 74 ± 5%, ACh 42 ± 8%*: % of total in fractions 6–9: Basal 23 ± 6%, ACh 58 ± 8%*, *P < 0.05 vs. Basal). Expression of p‐eNOS Ser1171 was localized in high‐density fractions and was not shifted by ACh stimulation (Fig. 4A). The distribution of iNOS, Cox1, and Cox2 was not different between basal and ACh stimulated ACh in aorta from WT LF (Fig. 4). Interesting, although actin was predominately localized in high‐density fractions under basal conditions, the percentage in high‐density fractions was increased with ACh stimulation (Fig. 4G, Basal 81 ± 6%, ACh 98 ± 1%*, * *P* < 0.05 vs. Basal).

**Figure 5:**
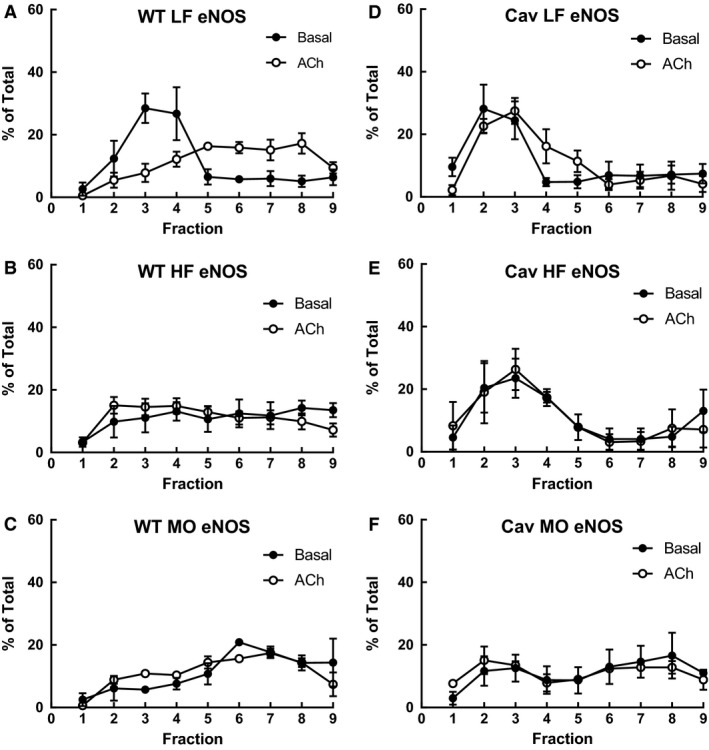
Expression of eNOS as a percent of total in low‐density fractions (1) to heavy density fractions (9) of aorta from WT (A, B, C) and cav‐1 KO mice (D, E, F) on LF, HF, or MO diet under basal conditions (closed circles) or following relaxation to ACh (1 *μ*mol/L, open circles). Mean ± SEM, n: WT: LF 5, HF 4, MO 3; cav‐1 KO: LF, HF, and MO 3 each.

The most striking effect of diet was a shift in eNOS localization in aorta from both HF and MO fed mice. eNOS was uniformly expressed throughout the gradient. In both HF and MO eNOS expression was reduced in low‐density fractions compared with LF Mice (% of total in fractions 2–5: Basal LF 74 ± 5%, HF 45 ± 9%*, MO 30 ± 6%*, *P < 0.05 vs. LF) despite persistent localization of cav‐1 to low‐density fractions (Fig. 5A–C). Similar to WT mice on LF, iNOS was predominantly in high‐density fractions and both Cox isoforms were expressed across the gradient similar to aorta from mice on LF diet (data not shown). In aorta from HF and MO mice stimulation with ACh had no effect on the distribution of eNOS (Fig. 5B and C), iNOS, Cox1 or Cox2 (data not shown). Thus, alterations in vascular fatty acid composition affect protein localization within lipid rafts that may define signaling and function.

To determine the role of cav‐1 and caveolae in effects of dietary fatty acids on localization of NOS and Cox in aorta we compared localization in non‐caveolar low‐density lipid rafts of aorta from cav‐1 KO mice under basal conditions and maximal relaxation with ACh. In LF cav‐1 KO mice under basal conditions, eNOS was again predominately localized within low‐density non‐caveolar lipid raft fractions, but in contrast to WT mice, ACh did not shift localization of eNOS to high‐density fractions (Fig. 5D). Similar to WT aorta, iNOS, Cox1, and Cox2 were either predominately in high‐density fractions or uniformly distributed throughout the gradient in aorta from LF cav‐1 KO mice and not affected by ACh (data not shown). HF diet and ACh had no effect on eNOS localization in aorta from cav‐1 KO mice (Fig. 5E). MO diet tended to reduce the percentage of eNOS in lipid raft fraction in Cav‐1 KO mice but the change was not significant and was not affected by ACh (Fig. 5D vs. F, % of total in fractions 2–5: LF 62 ± 11%, MO 42 ± 17%). Thus, in contrast to WT mice, eNOS localization in low‐density non‐caveolar lipid rafts in aorta from cav‐1 KO mice on normal LF diet was no longer shifted with ACh suggesting a critical role for cav‐1.

### Effect of dietary fatty acids on vascular responses in WT and Cav‐1 KO mice

**Figure 6:**
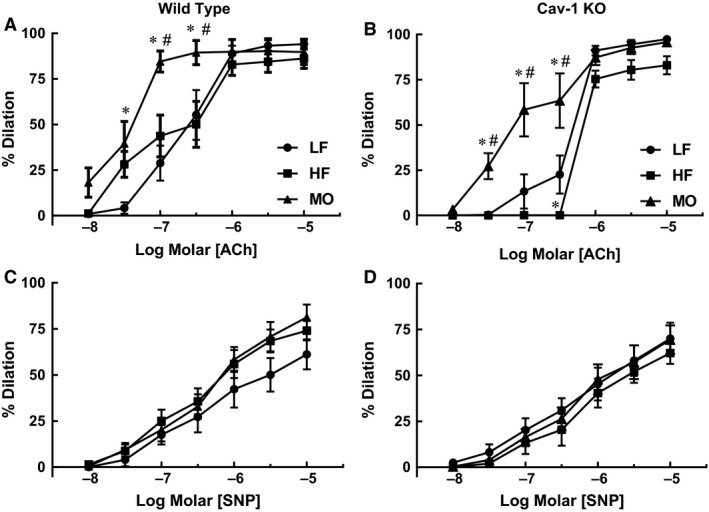
Responses to acetylcholine (ACh) and nitroprusside (SNP) of gracilis arteries from WT (A. and B. *n* = 7–11) and cav‐1 KO mice (C. and D. *n* = 8–9) on LF, HF and MO diets. Mean ±  SEM, *P < 0.05 versus LF, #*P* < 0.05 versus HF. Two‐way ANOVA with Tukey's for multiple comparisons.

To determine whether these changes in vascular fatty acid composition and membrane localization of signaling proteins affected vascular reactivity, we compared responses to ACh and SNP of gracilis arteries from WT mice on LF, HF, and MO diets. Diet had no effect on baseline diameters of gracilis arteries from WT mice (LF 113 ± 6 *μ*m, *n* = 9; HF 117 ± 7 *μ*m, *n* = 11; MO 125 ± 6 *μ*m, *n* = 8). ACh produced concentration dependent dilation of gracilis arteries from WT mice on all diets (Fig. 6A). HF diet did not affect dilation to ACh. However, MO markedly enhanced dilation of gracilis arteries to ACh (Fig. 6A). Although dilation to SNP tended to be enhanced in arteries from mice on both high‐fat diets, there was no significant effect of diet on responses to SNP (Fig. 6C). Thus, vascular responses to ACh were not affected by HF diet but substitution of a portion of the saturated fats with MO enhanced dilation of gracilis arteries to ACh.

We next examined effects of cav‐1 deficiency on vascular responses to ACh using cav‐1 KO mice. Baseline diameters of gracilis arteries from cav‐1 KO mice were not affected by diet although all tended to be greater than WT mice (LF 130 ± 8 *μ*m, *n* = 8; HF 145 ± 10 *μ*m, *n* = 8; MO 129 ± 4 *μ*m, *n* = 9). There was no difference in dilation to ACh in arteries from LF cav‐1 KO mice compared with LF WT (Fig. 6A and B). In contrast to WT mice, dilation to ACh was reduced in HF cav‐1 mice compared with LF (Fig. 6B). However similar to WT mice, replacing half of the saturated fat with n‐3 MO increased dilation of gracilis arteries from cav‐1 KO mice to ACh (Fig. 6B). Diet had no effect on dilation in response to SNP in cav‐1 KO mice (Fig. 6D). Thus, HF diet had a greater impact on responses to ACh in gracilis arteries from cav‐1 KO mice compared to WT mice. The beneficial effect of n‐3 PUFA MO to enhance dilation to ACh in gracilis arteries was independent of cav‐1.

**Figure 7:**
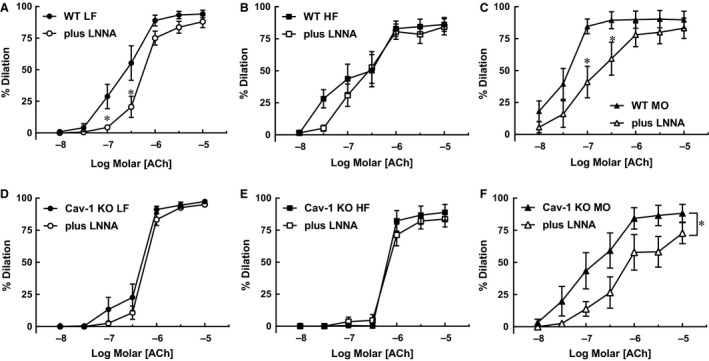
Responses to acetylcholine (ACh) before and after inhibition of NOS with nitro‐L‐arginine (LNNA, 100 *μ*mol/L) of gracilis arteries from WT mice on LF (A. *n* = 9), HF (B. *n* = 11) or MO (C. *n* = 7) diets and from cav‐1 KO mice on LF (D. *n* = 8), HF (E. *n* = 8) or MO (F. *n* = 8) diets. Mean ± SEM, *P < 0.05 versus control response. Two‐way ANOVA with Sidak's for multiple comparisons.

To determine whether dietary fatty acid saturation affects the contribution from NOS in responses to ACh, we examined effect of inhibition of NOS with LNNA (100 *μ*mol/L) in gracilis arteries from WT mice. In WT mice on LF diet, LNNA reduced ACh‐induced responses in gracilis arteries (Fig. 7A). In contrast in HF mice, inhibition of NOS had no effect on dilation to ACh (Fig. 7B). MO restored the contribution from NOS to ACh‐induced dilation of gracilis arteries (Fig. 7C). Thus, dietary fatty acid composition affected the contribution of NOS to responses to ACh in small gracilis arteries from WT mice.

To determine the contribution from cav‐1 and caveolae in NOS‐dependent dilation to ACh, we compared effects of LNNA on responses to ACh in arteries from cav‐1 KO mice. LNNA had no effect on dilation of gracilis arteries from LF cav‐1 KO mice to ACh which contrasts with WT mice where LNNA reduced dilation to ACh (Fig. 7A vs. D). Similar to WT HF mice, LNNA had no effect on responses to ACh in HF cav‐1 KO mice (Fig. 7E). Also similar to WT mice, MO restored the NOS‐component in ACh‐induced dilation since LNNA reduced responses in gracilis arteries from cav‐1 KO mice on MO (Fig. 7F). Thus, cav‐1 and caveolae deficiency only affected the contribution from NOS to responses to ACh in gracilis arteries from mice on LF diet. The effect of NOS inhibition was similar in WT and cav‐1 KO mice on HF and MO diets.

**Figure 8:**
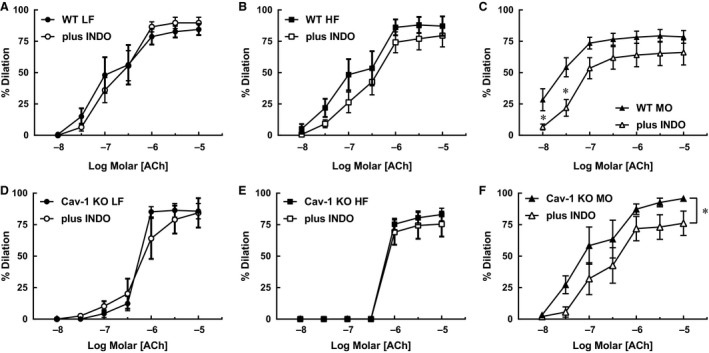
Responses to acetylcholine (ACh) before and after inhibition of Cox with indomethacin (INDO, 10 *μ*mol/L) of gracilis arteries from WT mice on LF (*n* = 7), HF (*n* = 11) or MO (*n* = 8) diets and from cav‐1 KO mice on LF (*n* = 6), HF (*n* = 9) or MO (*n* = 8) diets. Mean ± SEM, *P < 0.05 versus control response. Two‐way ANOVA with Sidak's for multiple comparisons.

The contribution from Cox in responses to ACh depends on tissue source, species and pathophysiology and is increased in obesity and diabetic models (Guo et al. 2005; Szerafin et al. 2006; Bagi 2009; Salvemini et al. 2013). We assessed responses to ACh in arteries from WT and cav‐1 KO mice on the different fatty acid diets following inhibition of Cox with indomethacin (10 *μ*mol/L). Indomethacin had no effect on dilation to ACh in gracilis arteries from WT or cav‐1 KO mice on either LF or HF diets (Fig. 8). In contrast, indomethacin significantly reduced dilation to ACh in arteries from both WT and cav‐1 KO mice on MO (Fig. 8). Thus, a diet enriched in n‐3 PUFA MO increased the contribution from Cox in responses of gracilis arteries to ACh independent of cav‐1 and vascular caveolae.

## Discussion

There were several findings in this study. First, equivalent calories from saturated fats only or a combination of saturated fats and n‐3 PUFAs produced similar increases in body weight though their effect on body fat differed. In wild‐type mice, n‐3 PUFA enriched diet did not increase body fat to the same degree as a diet high in saturated fats only. Cav‐1 deficiency diminished the effects of both high‐fat diets on body weight likely due to an inability to increase body fat. Second, dietary fatty acid composition affected the fatty acid composition of vascular tissue even in cav‐1 KO mice which showed modest changes in body weight and fat. The most prominent effect of the diet enriched in n‐3 PUFAs was an increase in the percentage of unsaturated fatty acids, in particular EPA (20:5) and DHA (22:6), and the unsaturation index in vasculature from both WT and cav‐1 KO mice. Third, high‐fat diet disrupted protein localization within low‐density cav‐1 containing lipid raft fractions. In aorta from LF mice, eNOS was localized to cav‐1 containing low‐density lipid rafts which were reduced with ACh. High saturated fats reduced the percentage of eNOS localized in cav‐1 containing low‐density fractions of aorta from WT mice. Adding n‐3 polyunsaturated fatty acids to the diet further reduced eNOS localization in low‐density lipid rafts and the distribution of eNOS in the sucrose gradient was no longer affected by ACh with HF or MO. Proteins localized in high‐density fractions or those not localized to a specific membrane component were not affected by dietary fatty acids. In the absence of cav‐1, eNOS was localized to non‐caveolar low‐density lipid rafts but localization was no longer affected by ACh. Fourth, the contribution from NOS in dilation to ACh was modified by dietary fatty acids. In wild‐type mice, high saturated fat had minimal effect on dilation to ACh but the NOS component observed in LF mice was absent. In the absence of cav‐1, NOS inhibition had no effect on responses of arteries from either LF or HF to ACh. This would suggest that cav‐1 is necessary for NOS activation by ACh with LF diet in gracilis arteries. However, with an increase in n‐3 PUFA, NOS activation was enhanced independent of cav‐1. Fifth, the role of Cox in responses to ACh was most prominent in both WT and cav‐1 KO mice on n‐3 PUFA MO enriched diet. Inhibition of Cox had no effect on ACh‐induced dilation of gracilis arteries from WT and cav‐1 KO mice on LF and HF diets. This suggests that the increase in the Cox component in ACh‐induced dilation with a diet high in n‐3 unsaturated fatty acids was also independent of cav‐1. In summary, although diet affects localization of eNOS within caveolar lipid rafts in WT mice, cav‐1 and caveolae alone do not define the contribution from eNOS in vascular responses to ACh. Diets enriched in n‐3 PUFA have the greatest impact on the contribution from Cox in responses to ACh, an effect that was also not dependent upon cav‐1 or localization within lipid rafts.

### Lipid rafts and dietary fatty acids

Following a meal, dietary fats are transported in the blood within large macromolecular complexes until they are hydrolyzed and released by lipoprotein lipase in vascular endothelium. Thus, vascular endothelium is one of the first cellular targets for dietary lipids. Long‐chain fatty acids derived from lipoproteins are precursors for complex plasma membrane lipids. The lipid composition of the plasma membrane is important because it is more than a simple boundary of the cell but serves as a multi‐domain structure which includes lipid rafts. Lipid rafts, small (10–200 nm) heterogeneous domains enriched in sphingolipid and cholesterol are dynamic centers regulating cell signaling (Yao et al. 2009). Formation of a specialized subset of lipid rafts, caveolae, is dependent upon the scaffolding protein caveolin to form flask‐shaped membrane invaginations. Caveolae do not form in the absence of caveolins but non‐caveolar lipid rafts persist. These non‐caveolar lipid rafts are more enriched in cholesterol and sphingolipids and may contain unique membrane proteins not found in caveolae (Yao et al. 2009). Deletion of cav‐1 prevents formation of caveolae in both endothelium and vascular smooth muscle (Hnasko and Lisanti 2003; Albinsson et al. 2007). Comparisons in the presence and absence of cav‐1 and caveolae allow us to separate differential effects of caveolae versus non‐caveolar lipid rafts.

If lipid rafts and caveolae are required for assembly and activation of receptors, signaling molecules and scaffolding proteins, then modifying the composition of these centers with cholesterol and dietary fatty acids may impact the regulation of cellular activity with potentially major implications. Diets high in n‐3 PUFAs reduce sphingomyelin and cholesterol content of caveolae and lipid rafts while increasing membrane n‐3 PUFAs (Ma et al. 2004; Adkins and Kelley 2010). In the present study, MO enriched diet increased levels of n‐3 DHA (22:6) and EPA (20:5) and unsaturation index of aorta. In model systems, both n‐3 and n‐6 PUFAs produce greater clustering of proteins within cholesterol‐rich lipid rafts compared with MUFA (Yaqoob and Shaikh 2010). Localization and activation of signaling proteins such as eNOS, normally abundant in caveolae, were suppressed in vascular tissue from mice on MO similar to colonic caveolae of mice fed n‐3 PUFAs (Ma et al. 2004; Seo et al. 2006). In our study, a high‐saturated fat diet shifted localization of eNOS from low‐density caveolar fractions to heavy fraction of unstimulated aorta in WT mice. Addition of MO to the high‐saturated fat diet further shifted localization of eNOS from low‐ to high‐density fractions. ACh stimulation only shifted eNOS from low‐density lipid raft fractions to the high‐density fraction in LF WT aorta where Ser1179 p‐eNOS was primarily localized. LNNA reduced ACh induced dilation of gracilis artery in LF and MO only. Thus, although dietary fatty acids affect vascular fatty acid content, the impact on vascular function did not correlate with changes in eNOS localization in lipid rafts.

### Mechanisms regulating eNOS activation

While lipid raft localization plays a role in eNOS regulation, additional mechanisms regulating activation include phosphorylation, protein‐protein interactions, fatty acid acylation, substrate availability as well as other post‐translational modifications (Michel et al. 1997; Fulton et al. 2002; Qian and Fulton 2013; Ramadoss et al. 2013). eNOS phosphorylation at multiple sites regulates activation with Ser1179 first identified as an important phosphorylation site by Akt, AMPK, CaMKinase II, and protein G (Qian and Fulton 2013). We found Ser1179 p‐eNOS primarily localized in high‐density fractions of both basal and ACh stimulated aorta from WT mice on LF. Ser1170 p‐eNOS was also localized to high‐density fractions in aorta of HF WT mice under basal conditions and with ACh induced relaxation (data not shown) suggesting that activated eNOS is primarily localized to non‐raft regions of plasma membrane. Despite a reduction in eNOS within low‐density lipid rafts in aorta from HF mice, LNNA did not reduce dilation to ACh. Inhibition of NOS reduced dilation to ACh only in arteries from LF‐ and MO‐fed mice. This would suggest that other regulators of eNOS activation including protein interactions, substrate availability or a combination of post‐translational modifications could be influenced by dietary fatty acids.

### Regulation of cyclooxygenase in obesity

The ability of vasculature to maintain vasodilator function in models of obesity and type 2 diabetes has been ascribed to a shift in the balance of NOS and Cox pathways (Guo et al. 2005; Szerafin et al. 2006; Bagi 2009; Salvemini et al. 2013). In animal models and human subjects with obesity, the contribution from Cox is enhanced to maintain responses to ACh (Guo et al. 2005; Szerafin et al. 2006; Bagi 2009; Salvemini et al. 2013). In obesity, increased Cox2 generates dilator prostaglandins most likely prostacyclin with little evidence that eNOS protein or phosphorylation is reduced compared with normal (Guo et al. 2005; Bagi 2009). An increased role for Cox2 in chronic, low‐level inflammatory models is well established but evidence that it is due to a change in protein expression is inconsistent (Bagi 2009). Obesity and type 2 diabetes are conditions with vascular inflammation. In our study, we did not find changes in expression of Cox1 or 2 in aorta with high‐fat feeding. Nor did inhibition of Cox affect responses to ACh in arteries from HF mice. Pharmacological inhibition of Cox only affected dilation to ACh in MO‐fed WT and cav‐1 KO mice. Thus, despite evidence a marked increased role for Cox in responses to ACh in arteries from mice‐fed n‐3 PUFA, mechanisms contributing to this shift are unclear. The increase in Cox mediated vasodilation did not involve cav‐1 but may be due, in part, to changes in Cox substrate (Salvemini et al. 2013).

### Effects of diet on vascular fatty acid composition

We previously reported effects of our diet strategy on serum and tissue fatty acids (Lamping et al. 2013). High saturated fat diet increased levels of glucose, cholesterol, insulin, and leptin but did not affect triglycerides and total free fatty acid levels. Substituting half of the saturated fatty acids with MO reduced cholesterol, insulin and leptin levels but not to levels in LF‐fed mice. Dietary fatty acids had a marked effect on both liver and muscle fatty acid composition (Lamping et al. 2013). Although high saturated fat diet increased hepatic 18:0 and 18:2 and reduced 16:0 levels, it had no effect on skeletal muscle fatty acid composition. The most prominent effect of n‐3 polyunsaturated‐enriched MO in liver and skeletal muscle was an increase in the levels of n‐3 20:5 EPA and 22:6 DHA and reduction in the level of n‐6 20:4. These changes in fatty acid saturation increased the unsaturation index. In this study, we found similar effects of dietary fatty acids on fatty acid composition of aorta. A high saturated fat diet had a modest effect on vascular fatty acid composition in WT and cav‐1 KO mice. The most striking effect of the MO enriched diet was an increase in the levels of DHA and EPA as well as the unsaturation index in both WT and cav‐1 aorta. It should be emphasized that our analysis of fatty acid composition was performed in whole aorta samples and does not specifically reflect levels in either endothelium or smooth muscle. It is possible that dietary fatty acids differentially affect fatty acid composition in endothelium versus smooth muscle which could not be distinguished with the current approach. However, the effect of dietary fatty acids on the contribution from Cox to vascular responses was independent of lipid rafts.

### Summary

We found that saturated and unsaturated dietary fatty acids affect eNOS localization in lipid rafts. While dietary fatty acids affect eNOS localization in caveolar lipid rafts in vasculature, this effect on eNOS localization within lipid rafts did not account for changes in the mechanisms underlying responses to ACh with high‐fat diets. Only an n‐3 enriched diet augmented the contribution from Cox to enhance dilation of peripheral vasculature to ACh. The ability of diets enriched in n‐3 PUFA to improve vessel function was independent of effects on eNOS or Cox localization within lipid rafts in the plasma membrane. These studies demonstrate that n‐3 PUFA markedly augment eNOS and Cox mediated vascular responses and support the notion that diets supplemented with n‐3 PUFA even in the presence of high levels of saturated fatty acids provide protection of vasculature in the presence of obesity.

## Conflict of Interest

None.
